# Proximal Sciatic Nerve Intraneural Ganglion Cyst

**DOI:** 10.1155/2009/810973

**Published:** 2009-12-22

**Authors:** Karin R. Swartz, Dianne Wilson, Michael Boland, Dominic B. Fee

**Affiliations:** ^1^Department of Neurosurgery, University of Kentucky, Chandler Medical Center, Lexington, KY 40536-0298, USA; ^2^Department of Pathology, University of Kentucky, Chandler Medical Center, Lexington, KY 40536-0298, USA; ^3^Department of Orthopedic Surgery, University of Kentucky, Chandler Medical Center, Lexington, USA; ^4^Department of Neurology, University of Kentucky, Chandler Medical Center, Lexington, KY 40536-0298, USA

## Abstract

Intraneural ganglion cysts are nonneoplastic, mucinous cysts within the epineurium of peripheral nerves which usually involve the peroneal nerve at the knee. A 37-year-old female presented with progressive left buttock and posterior thigh pain. Magnetic resonance imaging revealed a sciatic nerve mass at the sacral notch which was subsequently revealed to be an intraneural ganglion cyst. An intraneural ganglion cyst confined to the proximal sciatic nerve has only been reported once prior to 2009.

## 1. Introduction

Intraneural ganglion cysts are a rare condition. They are nonneoplastic, mucinous cysts within the epineurium of peripheral nerves. They most commonly affect the common peroneal nerve at the knee but can be seen elsewhere [1–3]. When the sciatic nerve is involved, it is classically an extension of a cyst from the distal sciatic branches, the common peroneal or tibial nerve [3–8]. We present a case of an individual with an isolated intraneural ganglion cyst of the proximal sciatic nerve; this is a rare entity, as only one such case was reported prior to 2009 [1, 9]. Poor recognition of this condition delayed definitive treatment. 

## 2. Case Presentation

A 37-year-old female with a history of pain in the left buttock and proximal posterior thigh, and endometriosis presented in April 2007 to the University of Kentucky Multidisciplinary Brachial Plexus Clinic for surgical evaluation of a painful mass in the left sciatic nerve. She had experienced sudden onset of left buttock and proximal posterior thigh pain eight years previously; this slowly progressed in intensity over time. The pain was exacerbated by certain positions such as sitting cross-legged on the floor. Since her symptoms initially fluctuated with her menses, they were ascribed to her known diagnosis of endometriosis. Hormonal therapy was employed and was effective initially. 

In 2001, the patient underwent magnetic resonance imaging (MRI) of the left thigh and spine which revealed a 2 × 3 cm enlargement, with edema, of the sciatic nerve at the left sciatic foramen, features consistent with uterine fibroids and unremarkable lumbar spine. MRI of her left hip and pelvis done in 2003 demonstrated resolution of the edema; the uterine fibroids were unchanged. In February 2007, she was reimaged due to worsening symptoms. MRI of the lumbar spine remained unremarkable; MRI of her left thigh and pelvis revealed a 2.9 × 3.4 × 3.2 cm lobulated mass incorporating the sciatic nerve at the sciatic notch; there was a bull's-eye pattern within several dilated tubular structures. The sciatic mass was hyperintense on T1—and T2 fat saturated—weighted images and hypointense on T2 inversion recovery images and had abnormal contrast enhancement. A substance within the mass had signal characteristics of blood or melanin. Also seen were atrophy and edematous changes with associated tendinopathy of the left gluteus minimus, gluteus medius, tensor fascia lata, obturator internus, and gluteus maximus muscles. There was no evidence for a labral tear; the cartilage overlying the femoral head and within the acetabulum and sacroiliac joints was similarly unremarkable. Nerve conduction studies (NCSs) of the left leg were significant for low-amplitude left sural sensory (5.4 *μ*V, normal >6.0 *μ*V) and left tibial motor (2.2 mV, normal >4.0 mV). The right sural sensory and tibial motor responses were normal, as were the left peroneal motor responses. Electromyography (EMG) of the left leg was significant for reinnervation changes in the left gluteus medius, maximus, and minimus muscles, and for reduced recruitment in the left long head of the biceps femoris and medial gastrocnemius; other left leg muscles were unremarkable. These abnormalities were consistent with her MRI: the NCS and distal EMG findings were consistent with a sciatic-nerve lesion; the proximal EMG findings reflected the atrophy and edematous changes seen in the gluteus minimus and medius muscles. Conservative measures to treat her pain including physical therapy, steroids, nonsteroidal anti-inflammatory agents, pregabalin, and narcotics proved unsuccessful. 

On presentation to our clinic, she was experiencing severe (10/10) pain in her left buttock, posterior left thigh, posterior left calf, and left foot. Certain positions, especially sitting on the floor (a frequent part of her job as a teacher) made it worse. When walking, she would experience intermittent loss of sensation in her left foot which would cause her to stumble and fall. At times, her left foot would be colder than the right. Her past medical history was significant for an exploratory laparotomy which confirmed the diagnosis of endometriosis. Her current medications included hydrocodone/acetaminophen, pregabalin, venlafaxine, and birthcontrol pills. Her family history, social history, and review of systems were significant for a negative family history for neurofibromatosis and the fact that she started to miss work as an elementaryschool teacher because of the pain.

**Figure 1:**
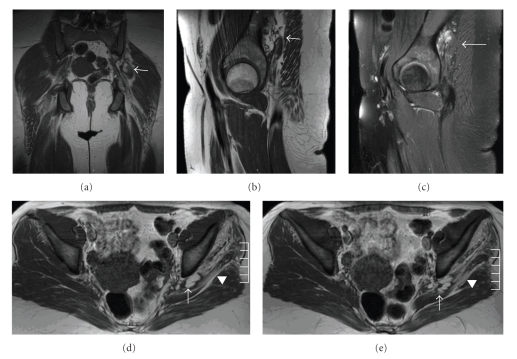
T1 (coronal (a), sagital (b), axial (d), and (e))—and T2 fat saturated (sagital (c))—weighted images of the left pelvis and hip. There is a 2.9 × 3.4 × 3.2 cm lobulated mass involving the sciatic nerve at the sciatic notch (arrow). It is hyperintense on T1 and T2 fat saturated images. Adjacent musculature, gluteus medius, and gluteus minimus are atrophic and have edematous changes (arrowhead).

On general physical and neurological examinations, notable findings included absence of obvious muscle atrophy, presence of pain-related limitation of the motor examination of the left leg (thigh extension and knee-flexion were at least 4 + /5 on the MRC rating scale), diminished sensation in the left foot to all modalities, and an antalgic gait. Somatosensory evoked potentials of the left sural and tibial nerves being normal. A repeat MRI of her left thigh and pelvis was essentially unchanged from the prior study (Figure 1). Radiographically, the lesion was felt to be a plexiform neurofibroma. 

**Figure 2:**
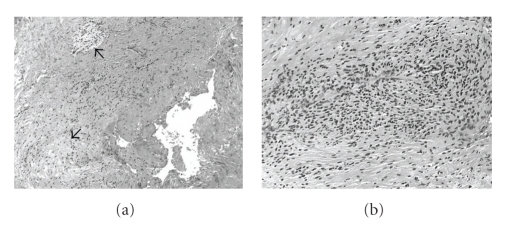
Hemotoxylin and eosin stained biopsy sections reveal (a) focal stromal myxoid changes (arrows), mild inflammatory infiltration, fibrosis, and (b) areas of synovial proliferation.

In June 2007, the patient underwent surgical exploration of the sciatic mass. Gross observation revealed a dark fluid, greenish-brown, which was encountered upon reflection of the piriformis, at the apex of the sciatic nerve in the leg. The sciatic nerve at the sciatic notch was enlarged and flattened; there was no discrete mass or cyst wall identified on gross inspection. Subsequent analysis of the fluid disclosed amorphous debris, 30,000 red blood cells/*μ*L, 120 white blood cells/*μ*L (57% neutrophils and 43% lymphocytes), and negative microbial stains or cultures. A nonconductive area of the nerve, identified by intraoperative stimulation, was biopsied and microscopically demonstrated a cyst wall with adjacent areas of synovial cell & perineural sheath proliferation, small collections of mucoid debris, foci of mild chronic inflammation, and myxoid degeneration (Figure 2). It is felt that the cyst seen on imaging was decompressed incidentally during the surgical exposure.

Postoperatively, the patient had improved strength and diminished pain in her left leg. She had acute worsening of pain four months after surgery, related to sitting cross-legged on the floor and having a child jump on her. An MRI of the pelvis and left thigh at that time showed postoperative changes in the sciatic nerve, no mass or lesion, and continued atrophy of gluteal muscles. At one year following surgery, she was markedly improved relative to her preoperative state. There was residual, position-only pain, maximum 4/10 intensity; she was requiring less medication for pain control.

## 3. Discussion

Intraneural ganglion cysts are a rare condition. Typically, they affect the common peroneal nerve at the fibular head but can be seen elsewhere [1–3]. The presented lesion was determined to be an intraneural ganglion cyst, given its presence within the sciatic nerve demonstrated on MRI and pathological findings from a biopsy of nonconducting area within the sciatic nerve; additionally there were no changes in the adjacent joints on MRI to suggest that this was a juxta-articular ganglion/synovial cyst. One etiological theory proposes dissection of synovial fluid into the epineurium of an articular branch nerve supplying a joint, with subsequent centripetal dissection into the parent nerve [3, 7, 10]. An alternative proposal is *de novo* cyst formation, either from embryonic remnants or from direct degeneration changes in the nerve [3, 5]. Our case does not support one etiology over the other; demonstrating a joint connection radiographically is difficult. Of the cases present by Spinner et al., the joint connection was not appreciated prospectively in any of the cases; they were only appreciated retrospectively [9].

The current case is exceptional not only for the duration of symptoms, but also for the involvement of the proximal sciatic nerve. Prior to 2009, there was only one reported intraneural ganglion solely involving the proximal sciatic nerve; this lesion also occurred at the sciatic notch [1]. In August 2009, Spinner et al. reported 4 cases of proximal sciatic nerve intraneural ganglion cysts, all demonstrating a joint connection in retrospective analyses of imaging [9]. In our case, because gluteal-muscle atrophy was demonstrated by EMG and on imaging, a proximal etiology was suspected and sought, but not demonstrated. The origin of the presented lesion was indeterminate radiographically and intraoperatively. The remainder of the visualized nerve was normal. The adjacent joints were unremarkable, making a labral cyst of the acetabulum unlikely [11–13]. It is possible that the adjacent muscle atrophy could be attributable to local effects secondary to the cystic lesion such as vascular compression and compromise (Figures 1(d) and 1(e)). On the other hand, muscle atrophy may be due to compromise of the nerve supply due to proximal extension of the sciatic intraneural ganglion cysts into the lumbosacral plexus [9]. It is possible that our patient had such an extension that was either not demonstrated on imaging or resolved prior to imaging. It is conjectural whether the patient's established practice of sitting cross-legged on the floor may have been a pathogenic factor, either by predisposing to epineurial dissection of synovial fluid or by direct injury to the sciatic nerve.

## 4. Conclusion

This case highlights the limitations of noninvasive testing in the setting of very unusual presentations. An isolated intraneural ganglion cyst of the proximal sciatic nerve was essentially an unreported condition prior to 2009. If recognized prior to surgery, effort must be made to determine if the intraneural ganglion cyst is extending into an articular branch nerve, so that this branch can be fully resected at surgery to decrease the risk of recurrence per Spinner and further our understanding of this rare condition.
